# Chemical and Biological Mechanisms Relevant to the Rescue of MG-132-Treated Neurons by Cysteine

**DOI:** 10.3390/antiox14020128

**Published:** 2025-01-23

**Authors:** Anna-Katharina Ückert, Ilinca Suciu, Anja Land, Anna-Sophie Spreng, Hannah Welte, Doreen Herzog, Michael Basler, Marcel Leist

**Affiliations:** 1 In Vitro Toxicology and Biomedicine, Chair Inaugurated by the Doerenkamp-Zbinden Foundation, University of Konstanz, 78457 Konstanz, Germany; 2 Department of Chemistry, University of Konstanz, 78457 Konstanz, Germany; 3 Konstanz Research School Chemical Biology (KoRS-CB), University of Konstanz, 78457 Konstanz, Germany; 4 Division of Immunology, Department of Biology, University of Konstanz, P1101 Universitätsstrasse 10, 78457 Konstanz, Germany; 5 Institute of Cell Biology and Immunology Thurgau (BITG), University of Konstanz, 8280 Kreuzlingen, Switzerland

**Keywords:** proteasome inhibition, cysteine, glutathione, antioxidants, neurotoxicity, transcriptome

## Abstract

Proteasome dysfunctions are observed in many human pathologies. To study their role and potential treatment strategies, models of proteasome inhibition are widely used in biomedical research. One frequently used tool is the proteasome inhibitor MG-132. It triggers the degeneration of human neurons, and several studies show protection from pathological events by glutathione or its precursors. It has therefore been concluded that glutathione protects cells from proteasome dysfunction. However, an alternative explanation is that MG-132, which is a peptide aldehyde, is chemically inactivated by thiols, and the apparent protection by glutathione from proteasome dysfunction is an artefact. To clarify this issue, we examined the chemical inactivation of MG-132 by thiols and the role of such reactions for neuroprotection. Using mass spectrometry and nuclear magnetic resonance spectroscopy, we found that MG-132 reacted with L-cysteine to form a stable end product and with glutathione to form an unstable intermediate. Using a cell-free proteasome inhibition assay, we found that high concentrations of L-cysteine can scavenge a substantial fraction of MG-132 and thus reduce proteasome inhibition. Glutathione (or N-acetyl-cysteine) did not alter proteasome inhibition (even at high concentrations). In a final step, we studied human neuronal cultures. We exposed them to MG-132, supplemented the culture medium with various thiols, and assessed intracellular L-cysteine concentrations. The transcriptome response pattern also indicated an inhibition of the proteasome by MG-132 in the presence of L-cysteine. We conclude that thiol concentrations that can be reached in cells do not inactivate MG-132 in pathological models. They rather act in a cytoprotective way as antioxidants.

## 1. Introduction

The rescue of neural cells by cysteine (Cys)-supplying compounds has been frequently reported, in particular, for neurodegeneration models related to Parkinson’s disease and triggered by the proteasome-inhibitor MG-132 [1,2,3,4,5,6,7,8]. It has been suggested that endogenous thiol supply, e.g., from astrocytes to neurons, may also attenuate neuronal proteotoxic stress [1]. The suggested underlying mechanisms range from an increase in cellular glutathione (GSH) levels to covalent binding and the scavenging of the toxic agent. The identification of the most relevant mechanism in a clinical setting is important for the design of potentially neuroprotective strategies applicable to human disease.

The proteasome is a multi-subunit protein complex responsible for protein degradation. Several subunits have proteolytic activities, which are described as chymotrypsin-like, caspase-like, and trypsin-like [9,10,11]. Proteasome activity is compromised in neurodegenerative disorders such as Parkinson’s disease [9,12,13]. Moreover, the pharmacological inhibition of the proteasome triggers the death of dopaminergic neurons in in vivo and in vitro models [14,15,16].

MG-132 was discovered as a specific proteasome inhibitor in the 1990s, and it was used to identify and clarify several fundamental functions of the proteasome in cell biology and immunology. It inhibits the chymotrypsin-like (β5) sites of the proteasome at low nM concentrations. The inhibition of caspase-like (β1) and trypsin-like (β2) activity sites also occurs at higher exposure levels [17,18]. The compound is cell-permeable and acts reversibly in cells, although it binds covalently to the catalytic proteasome subunits [19,20]. It has since been widely used to model neurodegeneration and several other pathologies.

N-acetyl-cysteine (NAC), GSH, Cys, and general thiol supply by astrocyte co-culture [21] are commonly used in the literature to rescue cells from MG-132. Various types of neuronal cells were protected by NAC [2,3,4,5,6,22]. Similar findings have been obtained for Cys or GSH supplementation by direct addition or when released from astrocytes [1,7,8,23].

LUHMES cells are human-derived, conditionally immortalized cells that exhibit a post-mitotic, neuronal phenotype upon differentiation. The mesencephalic neural precursor cells can be differentiated by shutting down the *myc* transgene [24,25]. They have been used in numerous studies to model Parkinson’s disease neuropathology [1,24,25,26,27,28,29,30,31,32,33,34,35]. They have also been used to investigate proteasome stress [1,23,33,34,36].

Proteasome inhibition in human dopaminergic LUHMES neurons leads to proteostatic stress [1]. Energy metabolism is compromised and oxidative stress occurs [33]. On a metabolic level, changes can already be seen after 3 h, and transcriptional changes occur mostly between 6 and 12 h. Protective counter-regulations of the cell itself are focused on GSH up-regulation and the synthesis of proteasome subunits. Neurons, treated with MG-132, can be protected by thiols released from co-cultured astrocytes or by the direct addition of Cys and GSH to the culture medium [1,33].

The exact nature of this protection is still under debate: it can either be attributed to the antioxidative effects of the thiols or it may be due to a covalent reaction with MG-132 [1]. MG-132 is a peptide aldehyde, and such aldehydes are known to react with thiols to form hemithioacetals [17,20,37]. Non-enzymatic reactions of aldehydes with thiols found in amino acids, peptides, and proteins have been described [38]. Moreover, Cys has been used in multiple instances to remove aldehydes from solutions [39,40,41,42]. The thiol–aldehyde reaction is also used to create hydrogels [43,44] and removable wound dressings [45]. Finally, various fluorometric detection methods for Cys are based on its reaction with an aldehyde probe [46,47]. Thus, it is possible that experimental artefacts are generated in any of the many model systems that use aldehyde drugs in the presence of thiols.

Therefore, we aimed here to determine whether MG-132 reacts covalently with thiols (Cys, GSH, and NAC) relevant for cell culture experiments, whether this reaction could occur under physiological conditions, and whether it may impact cell culture experiments during thiol intervention studies.

## 2. Materials and Methods

### 2.1. Materials and Chemicals

Dibutyryl cyclic adenosine monophosphate (dBcAMP), fibronectin, poly-L-ornithine hydrobromide (PLO), L-cysteine (L-Cys), glutathione (GSH), and tetracycline were purchased from Sigma (Steinheim, Germany). Epoxomicin was purchased from Enzo Life Sciences (Lörrach, Germany). MG-132 was purchased from SelleckChem (Köln, Germany). Recombinant human fibroblast growth factor 2 (FGF-2) and recombinant human glial cell-derived neurotrophic factor (GDNF) were purchased from R&D Systems (Minneapolis, MN, USA). All cell culture reagents were purchased from Gibco/Fisher Scientific (Hampton, NH, USA) unless otherwise specified.

### 2.2. NMR Measurements

All ^1^H-NMR spectra were acquired at T = 298 K by operating an 800 MHz Neo NMR spectrometer (Bruker Biospin, Ettlingen, Germany) equipped with a CP-QCI cryogenically cooled probe. Sample concentration of MG-132 was adjusted to 100 µM in 20 mM phosphate buffer containing 10% D_2_O (*v*/*v*) to maintain field lock. The observation of real-time NMR kinetics was initiated by adding 1 µL of a stock solution that comprises L-Cys or GSH (each possessing a concentration of 100 mM or 500 mM) to the solution of MG-132, leading to a twofold or tenfold molar excess of L-Cys or GSH regarding MG-132. In total, 400 one-dimensional ^1^H-NMR spectra were acquired, with 96 or 120 scans each. The spectral range between 7.2 ppm and 7.8 ppm was used for integration of resonance signals, exclusively reporting resonance signals of MG-132.

Equation (1) was then applied to determine the time constant, t1, characterizing the time-dependent change in resonance signals comprising MG-132 when L-Cys or GSH is added:

I = I0 × exp(−t/t1) + y,(1)

in which I represents the integral determined for all individual NMR spectra, I0 the initial integral, and y the final quantity of I reported upon the termination of the reaction.

### 2.3. MS Measurements

For LC-MS measurement, samples were separated on a liquid chromatography system (Infinity II 1260, Agilent Technologies, Santa Clara, CA, USA). Samples were resolved at a flow rate of 300 μL/min. A gradient of 5–100% of solvent B (100% ACN) over 2 min was used (solvent A: MQ). Column oven and sampler were set to 25 °C. Mass spectra were collected on a 6546 QTOF mass spectrometer (Agilent Technologies, Santa Clara, CA, USA) equipped with a dual AJS ESI source (Agilent Technologies, Santa Clara, CA, USA) and operated in positive mode with an acquisition rate of 1000 ms/spectra, 320 °C gas temperature, 8 L/min drying gas, 35 psi nebulizer, 350 °C sheath gas temperature, and 11 L/min sheath gas flow. Fragmentor voltage was set to 175 V, skimmer to 65 V, Vcap to 3500 V, and nozzle voltage was set to 1000 V. Data were recorded by MassHunter (version 10.1.62, Agilent Technologies, Santa Clara, CA, USA) and analyzed by MassHunter qualitative analysis (version 10.0, Agilent Technologies, Santa Clara, CA, USA). ESI TIC scan was extracted and integrated (integrator selection: Agile 2) from 0.1 to 0.5 min, and extraction data format was set to ‘Profile only’. Background was subtracted by subtraction of background spectra, and ESI scan data were exported as csv file. The respective peak areas were quantified in GraphPad Prism 8.

### 2.4. Proteasome Inhibition Assay

The assay was adapted from Basler and Groettrup (2012) [48]. Standard proteasome was purified from human erythrocytes as previously described [48]. In brief, proteasome inhibitors and antioxidants were diluted to 4× the indicated concentrations in substrate buffer (50 mM Tris–HCl, 25 mM KCl, 10 mM, NaCl, 1 mM MgCl_2_, and 0.1 mM EDTA at pH of 7.5) and pre-incubated for the indicated times at room temperature. The purified proteasome was diluted to 2 µg/mL in substrate buffer. One hundred µL of proteasome solution and 50 µL of inhibitor/antioxidant-solution were added to a black, flat-bottomed 96 wp and incubated for 30 min at 37 °C in the dark; for the negative control, 50 µL of substrate buffer was added instead of the inhibitor/antioxidant solution. Fifty µL of Suc-LLVY-AMC substrate (Bachem, Bubendorf, Switzerland) diluted in substrate dilution buffer were added to achieve a final concentration of 100 µM. Two hundred µL substrate dilution buffer containing 100 µM Suc-LLVY-AMC substrate was prepared as a background control. All conditions were assessed in triplicates. Plates were incubated at 37 °C in the dark, and fluorescence (ex: 351 nm/9 nm; em: 430 nm/20 nm) was measured after 30 min, 60 min, and 90 min. The average of the background control was subtracted from all measurements, and they were subsequently normalized to the negative control.

### 2.5. LUHMES Cell Culture

LUHMES cells were cultivated as described previously [24,49,50]. In brief, cells were grown in standard cell culture flasks pre-coated with 50 µg/mL PLO and 1 µg/mL fibronectin in water for at least 3 h at 37 °C. The maintenance culture was kept in proliferation medium consisting of advanced DMEM/F12 with 2 mM L-glutamine, 1× N2 supplement, and 40 ng/mL FGF-2. The cells were incubated at 37 °C with 5% CO_2_ and passaged three times a week when reaching 75–90% confluence. They were used up to passage 18. For differentiation, the medium was changed to differentiation medium consisting of advanced DMEM/F12 supplemented with 2 mM L-glutamine, 1 mM dBcAMP, 1 µg/mL tetracycline, and 2 ng/mL GDNF on day of differentiation 0 (d0).

### 2.6. Fluorescence Imaging

For morphological assessment of mature LUHMES neurons, precursor cells were pre-differentiated for 2 days in cell culture flasks pre-coated as described above and differentiation medium. Then, 45,000 cells per well were seeded in differentiation medium into standard 96-well plates pre-coated as described above. They were treated for 18 h on d6 and stained 45 min before imaging with 1 μM calcein-AM and 1 μg/mL H-33342 for 30 min at 37 °C and 5% CO_2_. Fluorescent imaging was performed (AxioObserver, Zeiss, Oberkochen, Germany) with ex/em wavelengths of 575 ± 25/640 ± 35 nm.

### 2.7. Quantification of Viability and Neurite Area

For the quantification of viability parameters, precursor cells were pre-differentiated for 2 days in cell culture flasks pre-coated as described above and differentiation medium. Then, 45,000 cells per well were seeded in differentiation medium into standard 96-well plates pre-coated as described above. They were treated for 18 h on d6 and stained 45 min before imaging with 1 μM calcein-AM and 1 μg/mL H-33342 for 30 min at 37 °C and 5% CO_2_. Then, neurite area (NA) and viability (V) were assessed as described previously [51,52]. Image acquisition was performed using an Array-Scan VTI HCS Reader (Cellomics, Pittsburgh, PA, USA) equipped with a Hamamatsu ORCA-ER camera. Ten fields per well were imaged with 2 channels at 20× magnification (2 × 2 pixel binning). Ex/em wavelengths of 365 ± 50/535 ± 45 nm were used for H-33342 detection in channel 1 and 474 ± 40/535 ± 45 nm were used for calcein detection in channel 2. Nuclei were identified in channel 1 depending on intensity, area, size, and shape. Their outlines were expanded by 3.2 μm to define a virtual cell soma area (VCSA). All calcein-positive pixels were defined as viable cellular structures (VCSs). The NA was automatically calculated by excluding the VCSAs from the VCSs. Furthermore, all nuclei co-localizing with VCSs were defined as alive.

### 2.8. TempOSeq Sample Preparation

For the transcriptomics experiment, cells were seeded in 96-well plates (Greiner, Kremsmünster, Austria) at a density of 60 k/90 µL/well. The proteasome inhibition was performed for multiple exposure times (0–18 h time course) using MG-132 at 100 nM on d6. For the rescue, several different compounds were tested: 1 mM GSH, 1 mM and 10 µM L-Cys, and 1 mM NAC. For the co-treatments, the rescue compounds were pre-incubated with MG-132 for 1 h before cell exposure. Additional samples were prepared for the L-Cys rescue, applied with a 1 h or 6 h delay after the MG-132 treatment. For the rescue experiments, total MG-132 exposure times were 3, 9, 12, 15, and 18 h. Each rescue condition had its own MG-132 control. In addition, a 24 h MG-132 condition served as control for cell death.

Samples were prepared by cell medium removal and addition of 33 µL of 1× Biospyder Enhanced Lysis Buffer to each well. The plates were then incubated at 37 °C for 10 min, then sealed (Corning, Corning, NY, USA), frozen at −80 °C, and shipped on dry ice. Each treatment was performed in 3 biological replicates, with 2 technical replicates (for untreated controls: 4 technical replicates). The whole transcriptome sequencing was conducted at Bioclavis (Biospyder Tech., Glasgow, UK) using the targeted TempO-Seq technology (panel v2.1: 22,533 genes). In a PCR step, the amplification of a 50 bp fragment for each gene was performed using target-specific probes, which introduced the sample tags. The mapping of counts to genes was performed by Bioclavis [53] using a reference library of all amplification products.

In a data pre-processing step, samples with a library size < 1 million were filtered out, resulting in the exclusion of 1 sample. Regardless of their count size, all genes were kept in the analysis. Read counts were normalized by division to the total number of mapped sample reads and multiplication by 106, yielding counts per million (CPM). The effect of normalization was checked by box and distribution plots, and no further outlier samples were identified. For each treatment, the differential gene expression (DGE) analysis was performed relative to the control group using the DESeq2 (R) implementation of the Wald test [54]. After multiple test correction using the Benjamini–Hochberg algorithm, significant transcriptomic changes (adjusted *p*-value < 0.05 and absolute fold change > 1.5) were identified. The complete statistical results are provided in Supplement_TempOSeq.xlsx.

### 2.9. Amino Acid Analysis

LUHMES cells were seeded on d2 into coated 10 cm dishes at a density of 9 Mio cells per dish in 10 mL of differentiation medium without dBcAMP and GDNF. They were treated as indicated and then harvested on d3. The cell culture medium was aspirated, and the cells were washed once with PBS. Nine hundred µL of ice-cold 50% (*v*/*v*) MeOH was added, and the cells were scraped off into a reaction tube. Another 400 µL of ice-cold 50% MeOH was added for washing and transferred into the same reaction tube. All samples were shaken for 30 min at 4 °C and 1440 rpm in an Eppendorf Thermomix (Hamburg, Germany). Then, they were centrifuged for 30 min at 4 °C at 20,000× *g*. The supernatant was transferred into a fresh reaction tube. The pellet was resuspended in 400 µL of ice-cold 50% MeOH, shaken for 20 min at 4 °C and 1440 rpm, and centrifuged for 20 min at 4 °C at 20,000× *g*. The supernatant was added to the previously collected supernatant, lyophilized in a SpeedVac concentrator, and reconstituted in 135 µL of 2% sulfo-salicylic acid containing 10 µM norleucin as internal control. The individual amino acids were separated and quantified using a Sykam S433 amino acid analyzer (Sykam, Fürstenfeldbruck, Germany) with a lithium-based anion exchange column (7 µm diameter, 10% cross-links, cat# 5125022) and post-column derivatization with ninhydrin. Elution was performed with buffers of increasing ion strength (buffer concentration 0.12–0.45 M) and pH (pH 2.9 → pH 12), as well as a temperature gradient. The ninhydrin–amino acid reaction products were quantified at 570 nm and 440 nm, depending on the amino acids. The peak areas were determined using ChromStar version 7.0 software (SCPA, Weyhe-Leehste, Germany) and converted into concentrations using defined standards.

### 2.10. Determination of Cell Volume

The volume of LUHMES cells was determined with two methods: (1) Proliferating cells were passaged as usual and then centrifuged into a 15 mL reaction tube. The reaction tube and pellet were visually compared to an identical tube filled with water. The water level was adjusted to the pellet height, and the volume of the water was recorded. Then, the cells were re-suspended and counted to determine the volume of a single cell: V = 1370 µm^3^. (2) Proliferating LUHMES cells were passaged as usual and seeded into coated 24-well plates. After a brief attachment period, brightfield images were taken, and the diameters (d) of 22 cells were measured. The following formula was used: Volume = 1/6πd^3^: Volume = 1625 µm^3^. The results were averaged to a volume of 150 ± 18 nL for 100,000 LUHMES cells.

### 2.11. Data Analysis

The methods for statistical analysis are indicated in the respective figure legends.

## 3. Results and Discussion

### 3.1. Prevention of Proteasome Inhibition by High L-Cysteine Concentrations

**Figure 1:**
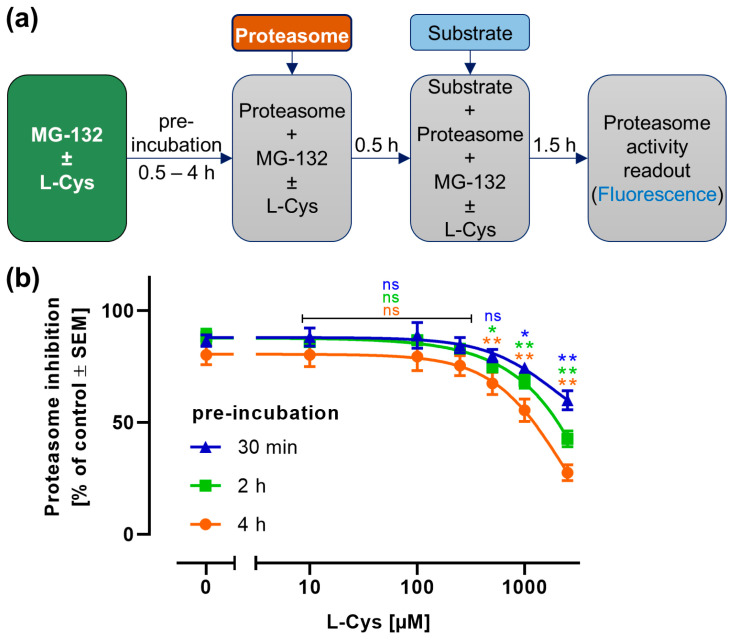
**The role of L-cysteine in the prevention of proteasome inhibition by MG-132.** A cell-free proteasome inhibition assay was performed using isolated proteasome and a fluorogenic substrate. (**a**) Treatment scheme: MG-132 (1 µM) was pre-incubated with L-cysteine (L-Cys) for the indicated time spans to allow for a potential chemical reaction. Then, isolated proteasome was added. Thirty min later, the substrate (Suc-LLVY-AMC) was added. The fluorescence was measured 90 min later. (**b**) The proteasome inhibition was quantified for pre-incubations of MG-132 with L-Cys for 0.5–4 h. All data are normalized to proteasome activity in the absence of MG-132, but in the presence of the respective L-Cys concentration. For statistical analysis, the L-Cys co-treated samples were compared to the samples treated with MG-132 only for the respective pre-incubation time, using a 2-way ANOVA followed by Dunnett’s multiple comparisons post hoc test (ns: not significant; *: *p* < 0.05; **: *p* < 0.001).

We were interested whether L-cysteine (L-Cys) would directly inactivate the proteasome inhibitor MG-132. As a model system, we used a cell-free proteasome preparation and measured its activity with a fluorescent substrate [48]. In this system, MG-132 (1 µM) efficiently blocked proteasome activity. In a variation of this experimental setup, MG-132 was pre-incubated with L-Cys to allow for a potential reaction to occur for 30 min, before the mixture was added to the proteasome preparation (Figure 1a). The addition of L-Cys alone had no significant effect on proteasome activity. To account for all small variations, all data on the proteasome inhibition of the MG-132/L-Cys co-treatments were normalized to the respective L-Cys control. The proteasome inhibition by MG-132 was significantly attenuated by L-Cys at concentrations ≥ 500 µM (Figure 1b). Prolonged pre-incubations of MG-132 with L-Cys (up to 4 h) led to a further attenuation of proteasome inhibition. The highest L-Cys concentration (2 mM) attenuated the effect of MG-132 by 70%.

As these experiments used a cell-free model, the observed inactivation of MG-132 cannot be attributed to cellular antioxidant pathways or to defense mechanisms that may be augmented by the supply of L-Cys to a deficient cell. It is more likely to result from a covalent binding of L-Cys to MG-132. We conclude from these data that there is a possibility for a direct inactivation of the proteasome inhibitor by added thiols.

### 3.2. Prevention of Cell Death and Neurite Loss by Thiols, Despite a Deregulated Transcriptome

**Figure 2:**
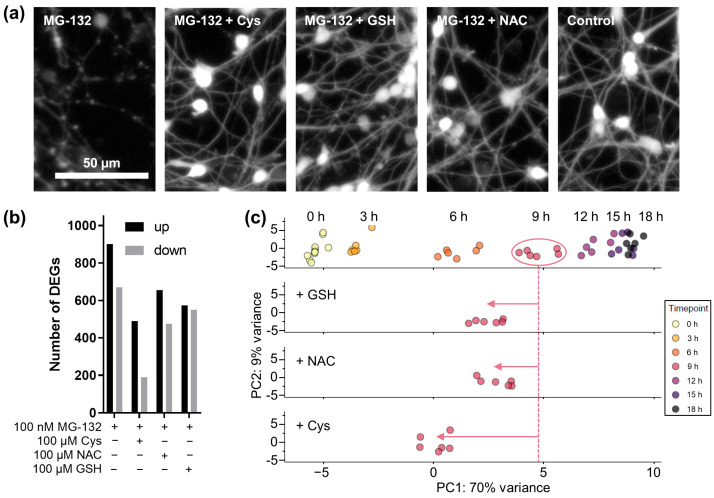
**Effect of thiol addition on transcriptome changes triggered by MG-132 in neurons.** (**a**) Differentiated LUHMES neurons (d6) were treated with MG-132 (100 nM) and the indicated thiol (100 µM; L-Cys: L-cysteine; GSH: glutathione; NAC: N-acetyl-cysteine). After 18 h, the cells were stained with calcein-AM and H-33342, and images were recorded on a high-content imaging system. Exemplary pictures of the calcein staining (= viable cellular structures) are shown. See Figure S1 for larger images. (**b**,**c**) A transcriptome analysis was performed on differentiated LUHMES neurons (d6), treated with MG-132 (100 nM) in the presence or absence of thiols (100 µM; L-Cys, GSH, NAC). (**b**) The number of differentially expressed genes (DEGs) was quantified. Full data sets on the time course are in Figure S3. Detailed data on all genes are in Supplement_DEG.xlsx. (**c**) A principal component analysis of the full transcriptome of all samples was performed. For better overview, samples are visualized in 4 sub-plots, which are all scaled in the same way. The upper plot shows samples exposed to MG-132 for 0–18 h. The 3 lower plots show samples exposed to MG-132 plus thiols for 9 h. The arrows indicate the relative shift along principal component 1 of the thiol-treated samples relative to the MG-132-only treatment (circled).

After observing the “protective” effect of L-Cys on the proteasome in a cell-free environment, we aimed to investigate more closely how L-Cys altered the response of live neurons to MG-132. LUHMES neurons were pre-incubated for 30 min with three different thiols: L-Cys, N-acetyl-cysteine (NAC), and glutathione (GSH). Then, MG-132 was added. After 18 h, cell viability and morphology were assessed. We confirmed our previous findings [1] that MG-132 induced neurite loss and cell death after 18 h (Figure 2a and Figure S2). Pre-treatment with all thiols (L-Cys, GSH, and NAC) prevented cell death and neurite loss (Figure 2a and Figure S2, with larger pictures in Figure S1), as demonstrated earlier [1]. All compounds were about equipotent, with a restoration of 50% viability by the addition of 25–30 µM thiol and at least 80% rescue by the addition of 100 µM of any of the thiols.

To investigate the status of treated cells in greater detail, we used a transcriptome analysis, as such an endpoint may indicate whether the direct biological effect of MG-132 was blocked within the live cells. We reasoned that the inactivation of MG-132 by thiols would not only increase neuronal viability but should also blunt cell stress responses triggered by proteasome inhibition. First, transcriptome de-regulation by MG-132 was recorded as the reference data set. Reference data on neurons treated with MG-132 alone were obtained as described [33]: LUHMES neurons were treated with MG-132 for 3 to 18 h. A principal component analysis (PCA) of the transcriptome data revealed a time-dependent deregulation of genes (Figure 2c). A quantification of the number of genes de-regulated by MG-132 alone is given in Figure S3a. Since cell death starts at 10–12 h [1], we expected that the samples taken after that time point would not be useful to show the effects of the thiols on non-dead cells. Therefore, we chose the 9 h time point (after the addition of MG-132) for obtaining the samples with thiol pre-incubation. The transcriptome analysis by PCA revealed that the thiols only had a mild (partial) effect on the transcriptional de-regulation (Figure 2b,c): The pre-incubations with the thiols resulted in a shift along principle component 1 (PC1) in the PCA plot. This means that they delayed the effects of MG-132. NAC and GSH caused a delay of about 2 h and L-Cys of about 4 h (when compared to 9 h samples treated with MG-132 alone). Notably, the thiols (L-Cys, GSH, and NAC) did not lead to a transcriptome de-regulation on their own (Figure S3b).

As we observed a clear transcriptome deregulation by MG-132 in the presence of thiols (despite the full morphological rescue of cells), we conclude that the thiols failed to de-activate MG-132 in the live neuron model. We suggest that the delay of the transcriptome response is more likely due to an unspecific (or indirect) cytoprotective effect of the compounds than to a chemical reaction with MG-132 within cells. The seemingly contrasting findings (MG-132 inactivation or no inactivation) in the two models used (cell-free vs. live cells) suggest, that the experimental details, such as the concentrations used, need closer investigation.

### 3.3. Reaction of MG-132 with L-Cysteine

**Figure 3:**
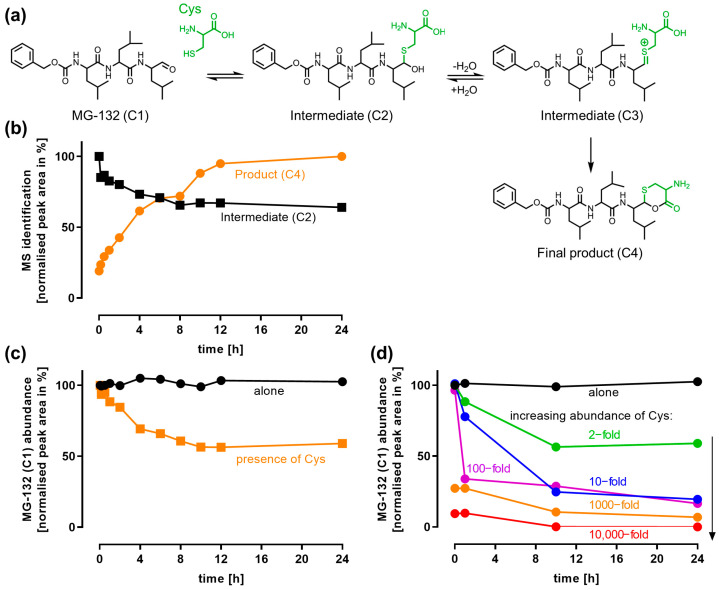
**Chemical reaction of MG-132 with L-cysteine.** (**a**) Proposed chemical reaction of MG-132 (C1) and L-cysteine (L-Cys, green) via 2 intermediates (C2, C3) to the final product (C4). (**b**) MG-132 (100 µM) and L-Cys (200 µM) were incubated in DMEM (cell culture medium) for 24 h and sampled at the indicated time points. The intermediate (C2) and the product (C4) were quantified via MS. (**c**) MG-132 was incubated in DMEM alone or in the presence of L-Cys (200 µM) for 24 h, during which MG-132 (C1) was quantified via MS. (**d**) MG-132 (100 µM) was incubated in DMEM with an increasing abundance of L-Cys (200 µM–1000 mM) over 24 h and quantified via MS.

Since we observed an inactivation of MG-132 in the cell-free proteasome assay, we aimed to understand the chemistry behind it. MG-132 is an aldehyde, and aldehydes are known to react with thiols to form hemithioacetals [17,20,37]. This reaction is reversible. However, we suggest that a stable cyclic product (C4) can be formed after water elimination (Figure 3a). This product is not expected to interact with the proteasome. To prove this theory, we performed mass spectrometry (MS) measurements on MG-132 co-incubated with various L-Cys concentrations. The reaction product (C4) accumulated over time (Figure 3b, Figures S4 and S5). The hemithioacetal intermediate (C2) was only found in a low amount, consistent with its unstable nature after its initial formation (Figure 3b).

The decline in MG-132 (C1) in the presence of L-Cys was assessed in more detail: within 10 h, about 50% of MG-132 was lost in the presence of 200 µM L-Cys (Figure 3c), while MG-132 alone was stable for 24 h under the same conditions. Increasing the abundance of L-Cys led to a faster depletion of MG-132: 10 mM L-Cys depleted MG-132 to 34% within 1 h. 1000 mM L-Cys completely depleted MG-132 within 10 h (Figure 3d).

We conclude that high L-Cys-concentrations (10–1000 mM) can effectively deplete MG-132 in cell-free environments via the proposed covalent reaction. This potential reaction should be kept in mind when performing pre-incubations of MG-132 and L-Cys. Within cells (or all culture media), L-Cys concentrations are usually << 1 mM. Extrapolating the active intracellular concentration of L-Cys from these nominal concentrations is quite challenging, since a range of factors are at play, such as the following: (1) autoxidation (e.g., L-Cys to cystine), (2) metabolic conversion (e.g., L-Cys to GSH), and (3) cellular uptake (thiols are not membrane-permeable), e.g., by the cystine transporter (slc7a11, Xc-System), which also plays a major role in the LUHMES cells used in this study [55]. Therefore, we analytically determined the intracellular L-Cys concentration after supplementation. We found that high enough concentrations (leading to hemithioacetal formation) are not reached in the cells (Figure S6): we found that after 5 h of thiol incubation, the intracellular L-Cys concentration (6 ± 1 µM) was increased to up to 260% of the control level. The intracellular GSH concentration (806 ± 21 µM) was increased to up to 210% of the control level. The levels of L-Cys observed here are relatively low, but they are well in agreement with most neurons requiring an L-Cys supply from astrocytes, and, e.g., LUHMES being highly sensitive to oxidative stress and the induction of ferroptosis [56]. For mature neurons (d6 LUHMES), an intracellular L-Cys content of 67 µM (1 nmol per 1 Mio cells) was found [1]. In liver tissue, L-Cys levels of 25 µM to 125 µM (assuming a cytosol volume of 800 µL per g liver) have been reported [57,58].

### 3.4. Dependence of Reaction Kinetics on L-Cysteine Concentrations

**Figure 4:**
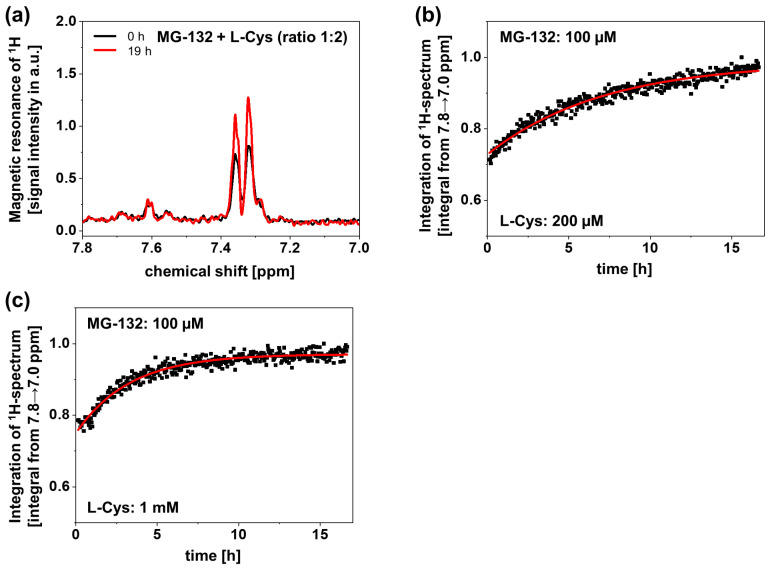
**NMR quantification of MG-132 reaction kinetics.** MG-132 (100 µM) was incubated with the indicated L-cysteine (L-Cys) concentrations for 19 h. ^1^H-NMR spectra were obtained at short intervals; each point represents one measurement. (**a**) The ^1^H-NMR spectra of MG-132 co-incubated with L-Cys (200 µM) taken after 0 h and 19 h are shown. (**b**,**c**) The integrals depicted in (**a**) were quantified and plotted over time to illustrate the reaction kinetics of MG-132 with L-Cys. Time points are given in black; the exponential fit is drawn in red.

To further quantify the reaction kinetics between MG-132 and L-Cys, nuclear magnetic resonance spectroscopy (NMR) was used. MG-132 (100 µM) and L-Cys were measured in fast intervals over 19 h. A specific window in the ^1^H-NMR spectrum (7.8–7.0 ppm) was chosen to quantify the reaction kinetics. The signal of MG-132 incubated alone did not change within the relevant timespan (Figure S7a). L-Cys (200 µM) had no signal in this interval (Figure S7b). The addition of L-Cys to MG-132 did not immediately change the signal either (Figure S7c); however, after 19 h of co-incubation, the signal changed significantly (Figure 4a). To properly quantify the reaction kinetics, the chosen interval was integrated at many time points (0–19 h), and the data points were fitted to an exponential function to determine the time constant of the reaction. The reaction was more than twice as fast with 1 mM L-Cys (Figure 4c) compared to 200 µM L-Cys (Figure 4b) (time constants of 3 h^−1^ vs. 7 h^−1^). These results confirm that a concentration- and time-dependent covalent reaction may occur between MG-132 and L-Cys at high L-Cys concentrations.

### 3.5. Reversible Depletion of MG-132 by Glutathione

**Figure 5:**
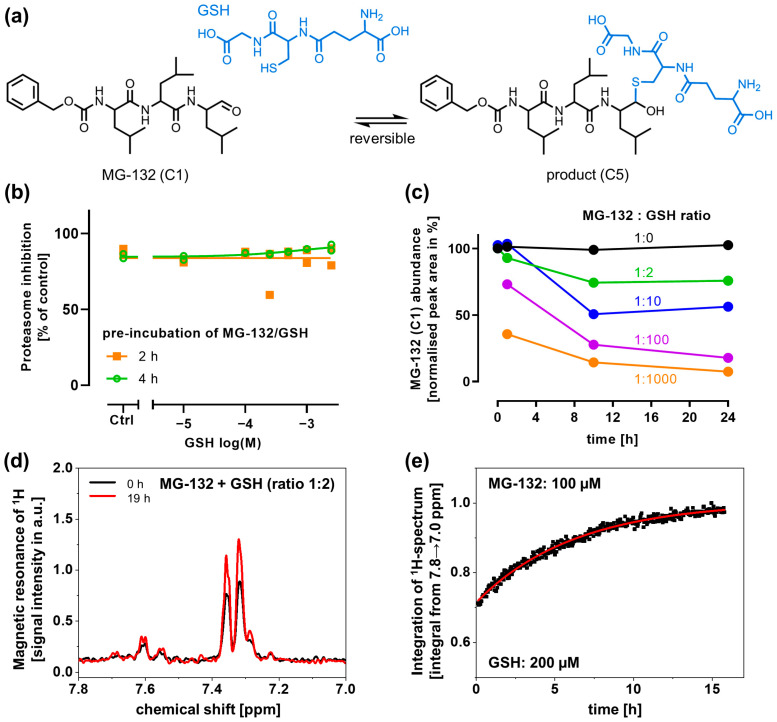
**Chemical reaction of MG-132 with GSH.** (**a**) Scheme of the likely chemical reaction of MG-132 (C1) and glutathione (GSH; blue) to the product (C5). (**b**) The proteasome inhibition by MG-132 (1 µM), pre-incubated with GSH, was quantified as described in Figure 1a. Data from 2 experiments are shown. All data are normalized to proteasome activity in the absence of MG-132, but in the presence of the respective GSH concentrations. (**c**) MG-132 (100 µM) was incubated in DMEM (cell culture medium) with increasing ratios of MG-132:GSH (GSH: 200 µM–100 mM) over 24 h, and MG-132 (C1) was quantified via MS. (**d**,**e**) MG-132 (100 µM) was incubated with GSH (200 µM) for 19 h. ^1^H-NMR spectra were obtained at short intervals. (**d**) The ^1^H-NMR spectra after 0 h and 19 h are shown. (**e**) The integrals depicted in (**d**) were quantified and plotted over time to illustrate the reaction kinetics. Time points are given in black; the exponential fit is drawn in red.

Next, we examined the potential reaction of MG-132 with GSH. We postulate that MG-132 reacts with GSH to form a hemithioacetal (product, C5). This reaction is analogous to the reaction of MG-132 with L-Cys. However, steric issues may prevent water elimination, and the formation of a stable cyclic product is not possible, as the carboxyl group of L-Cys in GSH is modified (by amidation with glycine; Figure 5a).

The proteasome inhibition assay was performed in the presence of MG-132 according to Figure 1. The prevention of MG-132-induced proteasome inhibition by GSH was not detected (Figure 5b). This suggests that GSH did not deplete MG-132 by an irreversible covalent reaction.

To follow up on this, we performed MS measurements of 100 µM MG-132 co-incubated with various GSH concentrations. We observed a depletion of MG-132 in a concentration- and time-dependent manner (Figure 5c; Figure S8 shows additional time points). At a very high concentration of 100 mM, GSH depleted MG-132 to 35% within 1 h; after 24 h, only 6% of MG-132 was left.

To confirm the chemical reaction and to obtain an insight into its kinetics, ^1^H-NMR measurements were performed. MG-132 (100 µM) and GSH were incubated over 19 h. GSH alone had no signal (in the quantified window; Figure S9a). It also had no immediate effect on the MG-132 signal (Figure S9b). After 19 h of co-incubation, the MG-132 signal changed significantly (Figure 5d). This change was quantified over time, and data were fitted to an exponential function (Figure 5e). The time constant (6 h^−1^) was similar to the one observed with L-Cys (7 h^−1^).

Taken together, these results suggest that GSH is indeed able to react with MG-132. However, the reaction appears to be reversible, as the proteasome is also inhibited by MG-132 in the presence of high concentrations of GSH. The findings suggest that when the proteasome is offered as an alternative binding partner to a GSH plus MG-132 mixture, it draws MG-132 from the reaction equilibrium and is inhibited.

### 3.6. Reaction of MG-132 with N-Acetyl-Cysteine to an Unstable Product

**Figure 6:**
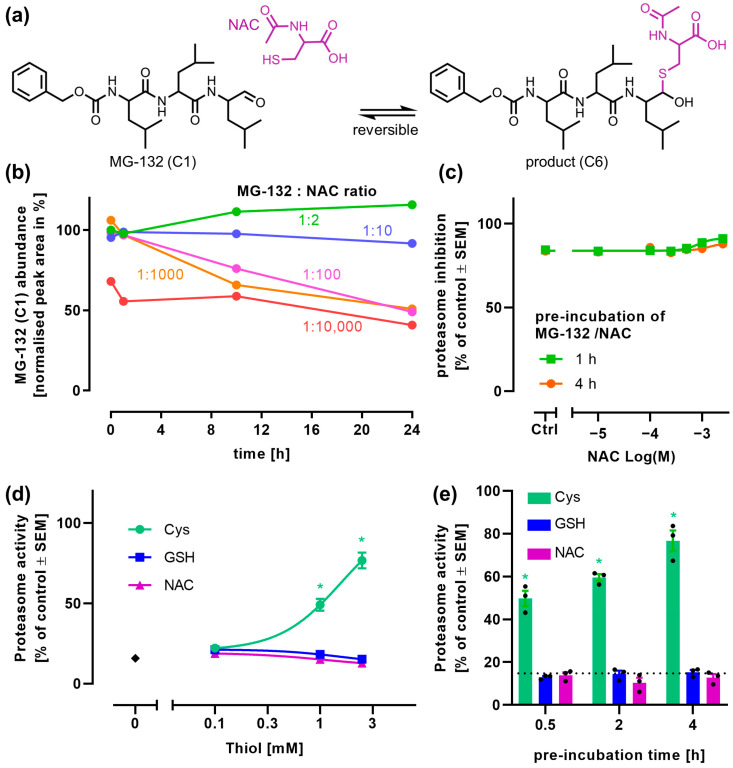
**Chemical reaction of MG-132 with NAC.** (**a**) Scheme of the likely chemical reaction of MG-132 (C1) and N-acetyl-cysteine (NAC; purple) to the product (C6). (**b**) MG-132 (100 µM) was incubated in DMEM (cell culture medium) with increasing ratios of MG-132:NAC (NAC: 200 µM–1000 mM) over 24 h, and MG-132 (C1) was quantified via MS. (**c**) Proteasome inhibition by MG-132 (1 µM), pre-incubated with NAC, was quantified as described in Figure 1a. Data are means ± SEM, n = 3. All data are normalized to proteasome activity in the absence of MG-132, but in the presence of the respective NAC concentrations. (**d**,**e**) Proteasome activity was assessed as in (**c**). All thiols were compared on the same plate in 3 separate experiments. The MG-132 only control is given in black. Data are means ± SEM, n = 3. (**d**) Proteasome activity after 4 h of pre-incubation of MG-132 (1 µM) with 0.1 mM, 1 mM, and 2.5 mM of the respective thiol. For statistical analysis, the thiol-co-treated samples were compared to the control samples treated with MG-132 only, using a 2-way ANOVA followed by Dunnett’s multiple comparisons post hoc test (*: *p* < 0.0001). (**e**) Proteasome activity after 30 min, 2 h, and 4 h of pre-incubation of MG-132 (1 µM) with 2.5 mM of the respective thiol (L-Cys: L-cysteine; GSH: glutathione; NAC). The dotted line indicates the proteasome activity after incubation with MG-132 (1 µM) alone. For statistical analysis, the thiol-co-treated samples were compared to the control samples treated with MG-132 only, using a 2-way ANOVA followed by Dunnett’s multiple comparisons post hoc test (*: *p* < 0.0001).

NAC has often been used as a rescuing thiol [2,3,4]. Therefore, we also investigated its possible reaction with MG-132. We postulate a reaction to a hemithioacetal (product, C6). Steric issues may or may not prevent water elimination and ring formation (Figure 6a).

Experimental data were required to decide whether NAC behaved similar to L-Cys or rather to GSH: MS was used to quantify MG-132 depletion in the presence of NAC (Figure 6b). To this end, 100 µM MG-132 was incubated with various concentrations of NAC. More than 10 mM of NAC was required to reduce the amount of MG-132. Even after 24 h, about 50% of MG-132 still remained.

A proteasome inhibition assay was performed as described in Figure 1 to determine whether NAC leads to a reduced inhibition of the proteasome. We observed that NAC failed to prevent proteasome inhibition (Figure 6c). Taken together, these results suggest that NAC behaves similar to GSH. In the absence of other binding partners, it can react with MG-132. However, once the proteasome is introduced as an alternative binding partner, MG-132 appears to be pulled from the reaction equilibrium (a reversal of the reaction with NAC).

To confirm the differential effects of L-Cys, GSH, and NAC on the prevention of proteasome inhibition within the same experimental setup, we repeated the proteasome inhibition assay in a way that all three thiols were prepared at the same time and compared side by side on the same plate (for the assay setup, see Figure 1). The data from this experiment confirmed that L-Cys increased proteasome activity at a concentration ≥ 1 mM. The other thiols did not show this effect, as expected from earlier findings (Figure 6d,e).

This confirms that L-Cys has unique properties amongst GSH precursors concerning the irreversible inactivation of MG-132. Ring formation after the elimination of water from the hemithioacetal is the most likely chemical explanation for our observations.

### 3.7. Re-Activation of the Proteasome by L-Cysteine After MG-132 Inhibition

**Figure 7:**
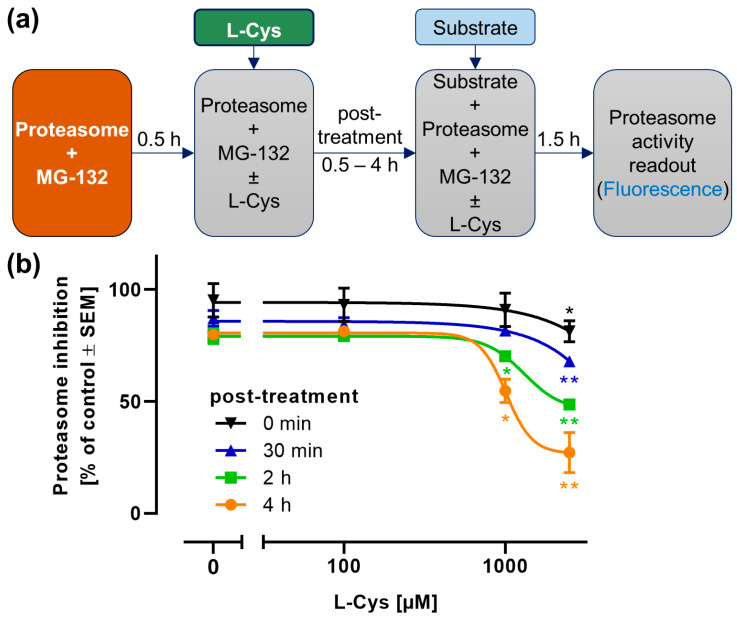
**Partial reactivation of the MG-132-inhibited proteasome by L-cysteine.** A cell-free proteasome inhibition assay was used as in Figure 1. (**a**) Treatment scheme: MG-132 (1 µM) was pre-incubated with the isolated proteasome for 30 min to allow for inhibition to occur. Then, L-cysteine (L-Cys) was added and incubated for the indicated time spans to allow a potential reactivation of the proteasome. Finally, substrate (Suc-LLVY-AMC) was added for 90 min before measurement of fluorescence. (**b**) The proteasome inhibition was quantified for reactivation times (post-treatment) with L-Cys for 1–4 h. Data were normalized to full proteasome activity (no MG-132) in the presence of the respective L-Cys concentrations. Data are means ± SEM, n = 3. For statistical analysis, the L-Cys-co-treated samples were compared to the control samples treated only with MG-132, using a 2-way ANOVA followed by Dunnett’s multiple comparisons post hoc test (*: *p* < 0.05; **: *p* < 0.001).

Having confirmed the potential of L-Cys to stably deplete MG-132 and to prevent subsequent proteasome inhibition, we were curious whether L-Cys could also restore proteasome activity after its inhibition by MG-132. To this end, we modified the proteasome assay from Figure 1 (Figure 7a). First, 1 µM MG-132 was incubated with the proteasome to allow for binding and inhibition. After 30 min, L-Cys was added for the indicated duration of time to possibly detach MG-132 from the proteasome. Then, the substrate was added and incubated for 90 min before measuring the proteasome activity as before.

L-Cys re-activated the proteasome time- and concentration-dependently (Figure 7b). Even when added together with the substrate, 2.5 mM L-Cys significantly reduced proteasome inhibition. When L-Cys was added to the inhibited proteasome for 4 h before the addition of the substrate, over 70% of the proteasome activity was restored.

These results show that L-Cys at high concentrations is able to break the reversible binding of MG-132 to the proteasome. We suggest that L-Cys would react with the detached MG-132 and thus remove the inhibitor irreversibly from the assay setup.

### 3.8. Prevention of Canonical MG-132-Induced Cell Stress Responses by Thiols

A brief summary of our findings up to this stage showed the following: (i) All three thiols may react with MG-132. However, this requires high concentrations (mM) compared to the cellular L-Cys concentration in the low µM range (Figure S6). Only the reaction with L-Cys appears to be irreversible. (ii) All three thiols prevented cell death at low extracellular concentrations (0.1 mM) and possibly low intracellular concentration increases (Figure S6). (iii) GSH and NAC had only a minor effect on the transcriptome response triggered by MG-132 (a 2 h delay), although they prevented cell death completely. (iv) L-Cys had a moderate (but far from complete) effect on the transcriptome response (a 4 h delay). These findings appear inconsistent with an inactivation of MG-132 by thiols within cells. It is more likely explained by an attenuation of the damage caused by proteasome inhibition at some downstream level.

We used a more detailed analysis of the transcriptome response to test our hypothesis that MG-132 does inhibit the proteasome and does not inhibit it in the presence of thiols, and that compounds like NAC (or L-Cys/GSH) protect cells from the downstream consequences of proteasome inhibition.

**Figure 8:**
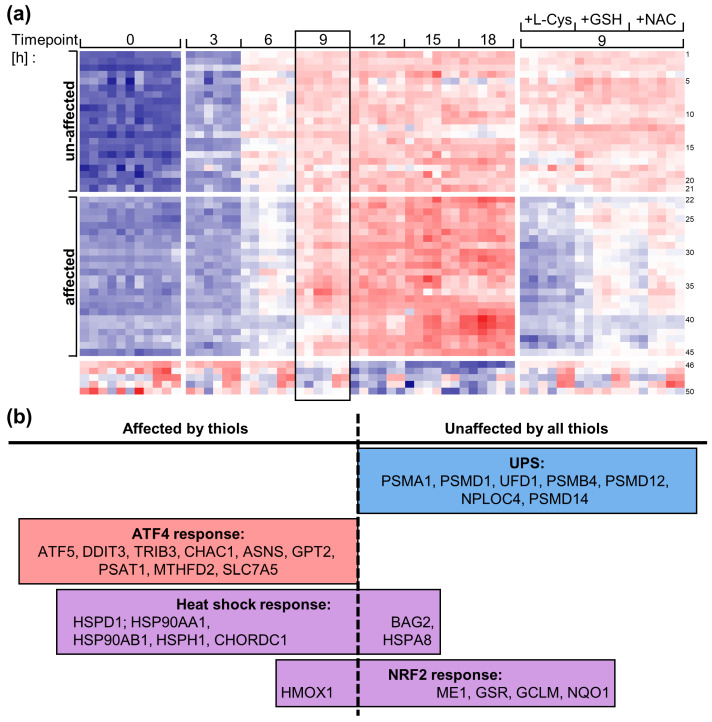
**Differential effects of thiols on gene expression changes by MG-132.** Differentiated LUHMES cells (d6) were incubated for 0–19 h with MG-132 (100 nM), as described in Figure 2 and Figure S10. The 9 h samples were in the presence or absence of thiols (100 µM; L-Cys: L-cysteine, GSH: glutathione, NAC: N-acetyl-cysteine). (**a**) The top 50 differentially expressed genes (DEGs) were identified. The color code indicates the row-wise z-score (full red: 1; white: 0; full blue: −2). The gene names (selected row numbers for orientation) are as follows, from top to bottom: *ME1* (1), *GSR*, *GCLM*, *NQO1*, *PSMA1* (5), *PSMD1*, *UFD1*, *PSMB4*, *PSMD12*, *NPLOC4* (10), *PSMD14*, *ANP32E*, *PLAA*, *PALM3*, *SCPEP1* (15), *BRF2*, *XPOT*, *VCP*, *CYB5R1*, *BAG2* (20), *HSPA8* (21), *HMOX1* (22), *HSPD1*, *HSP90AA1*, *HSP90AB1* (25), *HSPH1*, *CHORDC1*, *IARS1*, *GARS1*, *ATF5* (30), *DDIT3*, *TRIB3*, *CHAC1*, *ASNS*, *GPT2* (35), *PSAT1*, *MTHFD2*, *SLC7A5*, *SLC3A2*, *FLNC* (40), *TNFRSF10B*, *PPP1R15A*, *CBS*, *CBX4*, *ZFAND2A* (45), *PEG10*, *PCP4*, *MYH3*, *NEFM*, *ABRACL* (50). The genes were classified as “affected” or “un-affected” by thiols, depending on whether the co-treatment with thiols significantly reverted the MG-132-induced deregulation (box) towards the untreated expression level. (**b**) Most of the genes from (**a**) fall into 4 groups: ubiquitin–proteasome system (UPS), ATF4 response, heat shock response, NRF2 response. Gene groups unaffected by thiols are given in blue boxes, gene groups fully affected by thiols are given in red boxes, gene groups partially affected by thiols are given in purple boxes. The proteasome is still inhibited in the presence of thiols, which is indicated by the UPS and NRF2 response also being (mostly) unaffected by thiols. Downstream damage pathways (ATF4 and heat shock response) are, however, attenuated by thiols, which explains the rescuing effect seen morphologically (Figure 2).

LUHMES neurons were treated with 100 nM MG-132 and the indicated thiols (100 µM; see Figure 2). The 50 transcripts most affected by MG-132 were classified into those affected or unaffected by thiols (the top 100 deregulated genes are given in Figure S10; the full gene list with categories can be found in Supplement_DEG.xlsx). The L-Cys treatment had the most pronounced effect in preventing the MG-132-induced transcriptome changes, but on the whole, the regulation pattern by thiols was relatively similar (Figure 8a).

The regulation of genes indicative of a disturbance of the ubiquitin–proteasome system (UPS) was not affected by thiols (Figure 8b). Such transcripts code for proteasome units such as *PSMA1* or *PMSD1*. Their up-regulation is a cellular response to strongly lowered proteasome activity. Thus, they are generally considered an indication of a proteasome dysfunction (or in this case of proteasome inhibition by MG-132). As thiols did not change this dysregulation, we concluded that the proteasome was inhibited in thiol-treated cells.

The cell stress response driven by the oxidative stress transcription factor NRF2 was generally unaffected by thiols (Figure 8b). Proteasome inhibition is known to activate NRF2 [1,59,60], and thus, an upregulation of NRF2-dependent transcripts is expected, even though thiols provide protection from downstream damage events [1,60].

The MG-132 regulated genes affected by L-Cys (and other thiols) are typical indicators of the ATF4 stress response (Figure 8b). The ATF4 response is an archetypical indicator of ER stress [61]. Recently, ATF4 has also been recognized as a mediator of the mitochondrial stress response [62,63]. This can be a downstream consequence of proteasome inhibition [33], and it may be attenuated by thiols, even if the initial trigger (dysfunctional proteasome) remains. The same applies to the heat shock response, which was partially modified by cytoprotective thiols.

To sum up, the transcriptome study was used as a multidimensional intracellular probe to shed light on cell biological functions in the combined presence of thiols and MG-132. The readout pattern showed that the proteasome remained inhibited in the presence of thiols. At the same time, the transcriptome response showed that downstream stress responses, triggered by proteasome inhibition (ATF4 and heat shock response), are attenuated by thiols. Thus, the cells are rescued by, e.g., L-Cys, as it acts as a cellular stress modifier (antioxidant) and not by attenuating proteasome inhibition.

## 4. Conclusions

Our study design involved three levels of complexity: (i) studies of chemical reactions (in very simple environments), (ii) studies of enzymatic activity (the proteasome) and its modification in a cell-free, medium complex environment, and (iii) studies on cellular reactions in the presence of MG-132 and L-Cys. Our conclusion from the comparison of the many studies is that MG-132 does NOT react with thiols in a complex cellular environment, given the concentrations that may be reached in such studies.

On the second complexity level (cell-free), we demonstrate that L-Cys can irreversibly inactivate MG-132 at high concentrations. Using the first complexity level (chemistry), we identify the reaction product formed from L-Cys and MG-132. We also show that this chemistry does not work for GSH or NAC. The cytoprotection by GSH and NAC is therefore not likely to be related to a chemical inactivation of MG-132. We suggest that it is rather a consequence of their antioxidative effects on neurons. The cellular L-Cys concentration was found to be in the 10 (control)–25 (L-Cys supplemented) µM range (relevant for complexity level 3). This is 100× lower than the L-Cys concentrations that led to MG-132 inactivation in this study. Such conclusions were possible due to the experimental design at several complexity levels and exploring a large matrix of concentrations and times. An intracellular inactivation of MG-132 or a re-activation of the proteasome in cells is therefore considered highly unlikely. The findings have broad implications, as MG-132 has been used widely for neurodegeneration modelling (see Figure S11 for >20 examples). Such models are not designed to elucidate the etiology of diseases, but they are considered useful to study the pathological consequences downstream of proteasome inhibition, and they appear to be useful to test intervention strategies. In the context of choosing and constructing models (like exposure to MG-132), it may be useful to consider different disease phases. While many chronic diseases have an unknown trigger/primary pathogenesis, their propagation often involves common events, such as disturbed proteostasis, mitochondrial failure, and oxidative stress. It is widely believed that the prevention of such secondary propagation mechanisms can be useful, which forms a basis for drug development. This is the reason that such models keep being used and developed. Our research falls into this area. The data we provide suggest that thiol antioxidants work by a biological mechanism (rather than a chemical artefact), and that it is worth following this research line for neuroprotection.

For instance, our transcriptome study demonstrated an inhibition of the proteasome in the presence of thiols. Downstream damaging pathways (ATF4 and heat shock response) were, however, attenuated, which accounts for the rescue we observed.

These results confirm that the astrocyte- and thiol-mediated rescue of proteotoxic and oxidative stress induced by MG-132 in LUHMES neurons seen by Gutbier et al. was indeed due to the antioxidative effects of the thiols (GSH and L-Cys) and not due to a chemical inactivation of MG-132 [1]. As there are a lot of ongoing research studies concerning the beneficial/pharmacological use of thiol antioxidants, it is important to clarify whether the observed effects are genuinely due to antioxidant effects on a stressed model system (possibly transferable to the human situation), or whether the thiols basically destroy the basis of the model (by a simple chemical reaction). Our study indicates that previous and future experiments performed with rescuing thiols can be considered reliable and artefact-free.

However, the data from our study suggest that the inactivation of MG-132 by L-Cys should be kept in mind, when pre- or co-incubating MG-132 with high concentrations of L-Cys. Chemical interference is unlikely to be relevant when L-Cys is added to an experimental model after MG-132, at least as far as cellular or tissue systems are considered. A more general recommendation derived from our study is that a potential reaction with L-Cys needs to be considered when other toxicants or pharmacological tools containing aldehyde moieties are used.

## Data Availability

Data available upon request.

## Supplementary Materials

The following supporting information can be downloaded at: https://www.mdpi.com/article/10.3390/antiox14020128/s1: Supplement_DEG.xlsx, Supplement_TempOSeq.xlsx, and Supplement_Figures.docx. References [64,65,66,67,68,69,70,71,72,73,74,75,76,77,78,79,80,81,82] are cited in the supplementary materials.

## Abbreviations

The following abbreviations are used in this manuscript:

ATF4	Activating transcription factor 4
bp	Base pair
Cys	Cysteine
CPM	Counts per million
dBcAMP	Dibutyryl cyclic adenosine monophosphate
d	Diameter
DEG	Differentially expressed genes
DGE	Differential gene expression
dx	Day x of differentiation
em	Emission
ex	Excitation
FGF	Fibroblast growth factor
GDNF	Recombinant human glial cell-derived neurotrophic factor
GSH	Glutathione
L-Cys	L-Cysteine
LC-MS	Liquid chromatography–mass spectrometry
MS	Mass spectrometry
NA	Neurite area
NAC	N-acetyl-cysteine
NMR	Nuclear magnetic resonance
NRF2	Nuclear factor erythroid 2-related factor 2
ns	Not significant
PCA	Principal component analysis
PC#	Principle component #
PLO	Poly-L-ornithine hydrobromide
UPS	Ubiquitin-proteasome system
V	Viability
VCSA	Virtual cell soma area
VCSs	Virtual cellular structures
