# Supplementary material for: Chemical and Biological Mechanisms Relevant to the Rescue of MG-132-Treated Neurons by Cysteine
Source: Antioxidants (Basel). 2025 Jan 23;14(2):128. doi: 10.3390/antiox14020128 (PMC11851368; doi:10.3390/antiox14020128)
Supplement: Supplementary file 1 [file antioxidants-14-00128-s001.zip › Supplement_Figures_Ückert et al.pdf]

Supplementary information to:

Chemical and biological mechanisms relevant to the rescue of MG-132-treated neurons by cysteine

Anna-Katharina Ückert, Ilinca Suciu, Anja Land, Anna-Sophie Spreng, Hannah Welte, Doreen Herzog, Michael Basler, Marcel Leist

Table of Contents

Figure	Page	Title
S1	2	The effect of thiol addition on the morphology of MG-132 treated mature neurons
S2	3	The effect of thiol addition on the viability of MG-132 treated mature neurons
S3	4	The effect of thiol addition on transcriptome changes in MG-132 treated mature neurons
S4	5	Quantification of the intermediate (C2) and the product (C4) of the reaction of MG-132 with L-cysteine in water
S5	6	Spectra of MG-132 (C1), intermediate (C2) and product (C4) of the reaction of MG-132 and L-cysteine in DMEM medium
S6	7	Intracellular concentration of L-cysteine, glutathione, methionine and histidine after thiol incubation
S7	9	^{1}H-NMR-spectra of MG-132 and L-cysteine controls
S8	10	Quantification of MG-132 (C1) in the presence or absence of GSH over the course of 24 h
S9	11	^{1}H-NMR-spectra of MG-132 and GSH
S10	12	Differential effects of thiols on gene expression changes by MG-132 (Top 100 DEGs)
S11	14	Studies utilizing MG-132 to model neurodegeneration
	16	Supplementary references

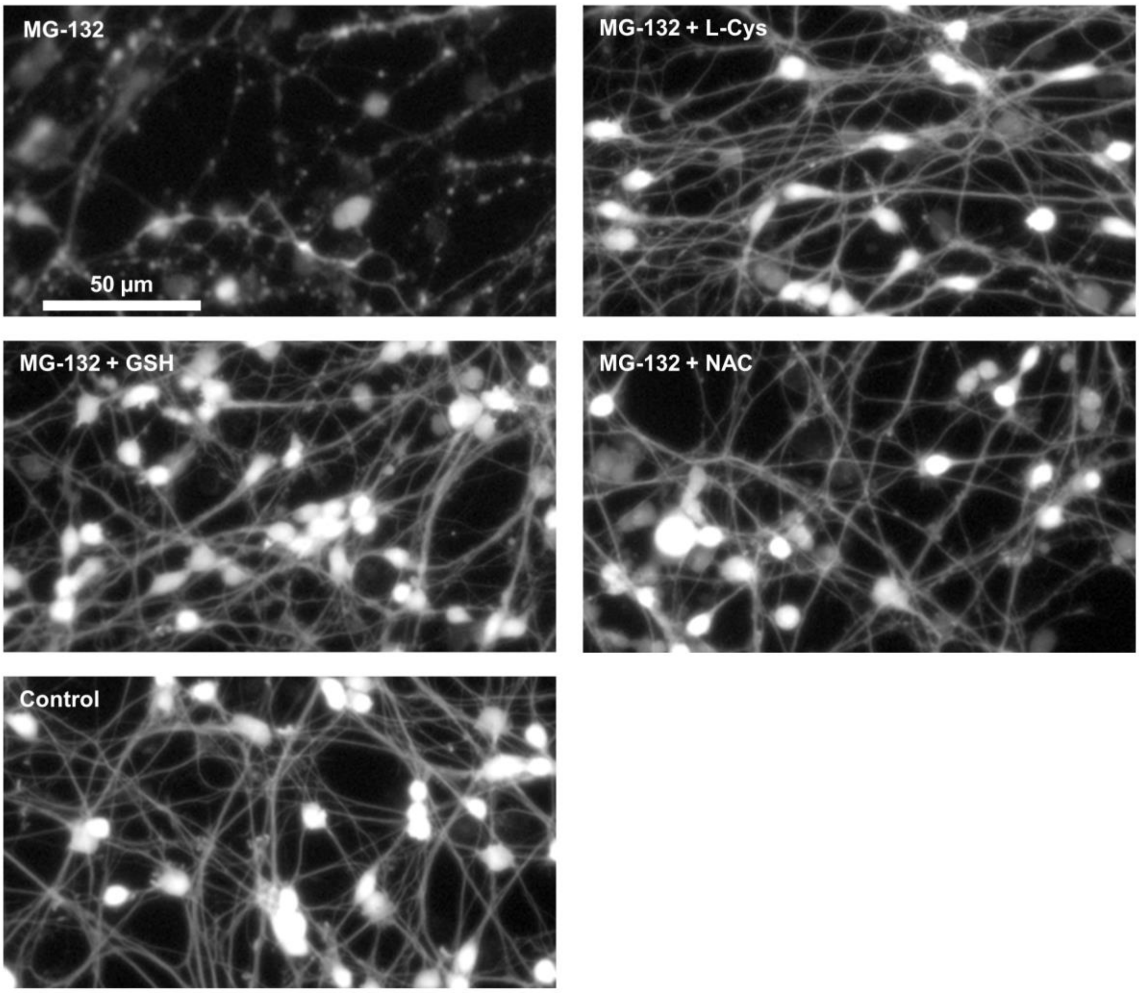

Figure S1: The effect of thiol addition on the morphology of MG-132 treated mature neurons

Differentiated LUHMES neurons (d6) were treated with MG-132 (100 nM) and the indicated thiols (100 µM; L-Cys: L-cysteine; GSH: glutathione; NAC: N-acetyl-cysteine). After 18 h, the cells were stained with calcein-AM and H-33342. Images were recorded on an automated high-content imaging system, as described earlier [1,2]. The data fully confirm earlier data. Evaluation of the experiment with a fully automated imaging algorithm is shown in Figure S2.

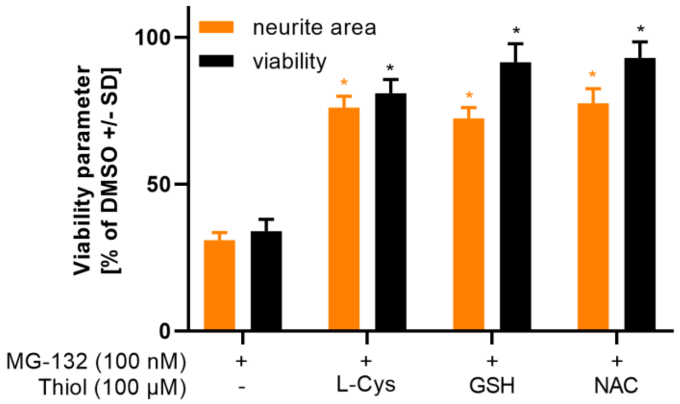

Figure S2: The effect of thiol addition on the viability of MG-132 treated mature neurons

Differentiated LUHMES neurons (d6) were treated with MG-132 (100 nM) and the indicated thiols (100 µM; L-Cys: L-cysteine; GSH: glutathione; NAC: N-acetyl-cysteine). After 18 h, the cells were stained with calcein-AM and H-33342, and images were recorded on a high-content imaging system. Neurite area and viability were quantified as described previously [3]. For statistical analysis, the co-treated samples were compared to the MG-132-only-treated samples, using a 2-way ANOVA followed by Dunnett's multiple comparisons post hoc test (*: $p < 0.001$).

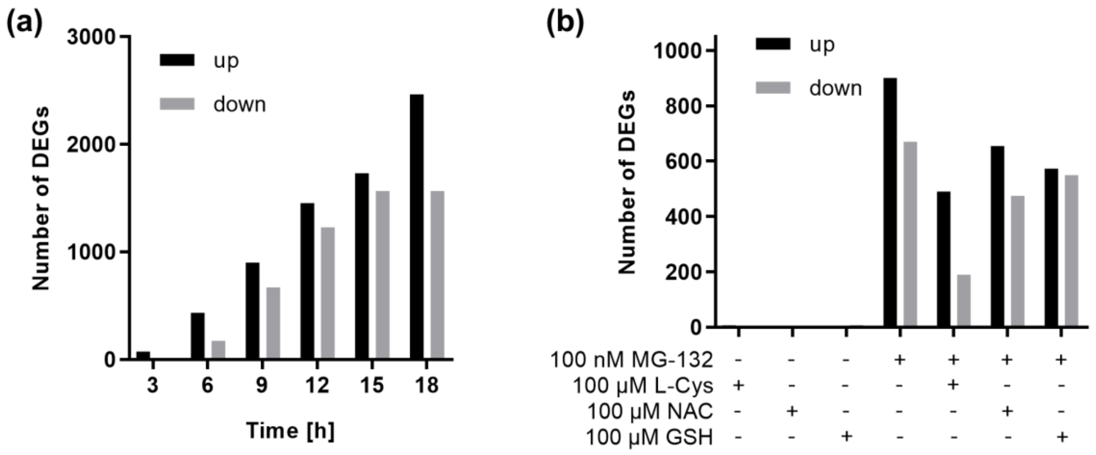

Figure S3: The effect of thiol addition on transcriptome changes in MG-132 treated mature neurons

A transcriptome analysis was performed on differentiated LUHMES neurons (d6), treated with MG-132 (100 nM) in the presence or absence of thiols (100 µM; L-Cys: L-cysteine; GSH: glutathione; NAC: N-acetyl-cysteine). The experiment was performed 3 times independently. Differentially expressed genes (DEGs) were determined statistically as described in the methods part, or in Fig 1. Separate information is given on up-regulated transcripts (up) and down-regulated transcripts (down=). (**a**) Neurons were treated only with MG-132 for up to 18 h. Samples were taken at the indicated time points. (**b**) Neurons were treated with MG-132 and/or thiols, as indicated.

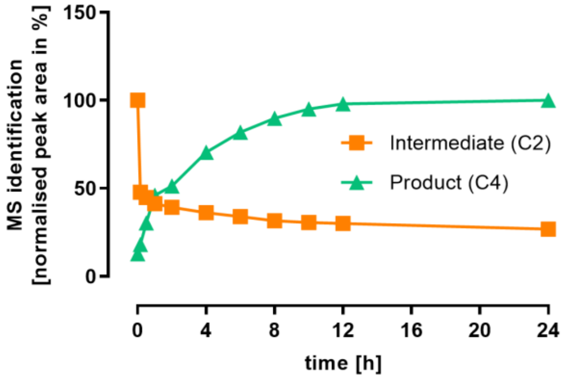

Supplementary Figure S4: Quantification of the intermediate (C2) and the product (C4) of the reaction of MG-132 with L-cysteine in water

MG-132 (100 µM) and L-Cys (L-cysteine; 200 µM) were incubated in water for 24 h, and sampled at the indicated time points. The intermediate (C2) and the product (C4) were quantified via mass spectrometry (MS).

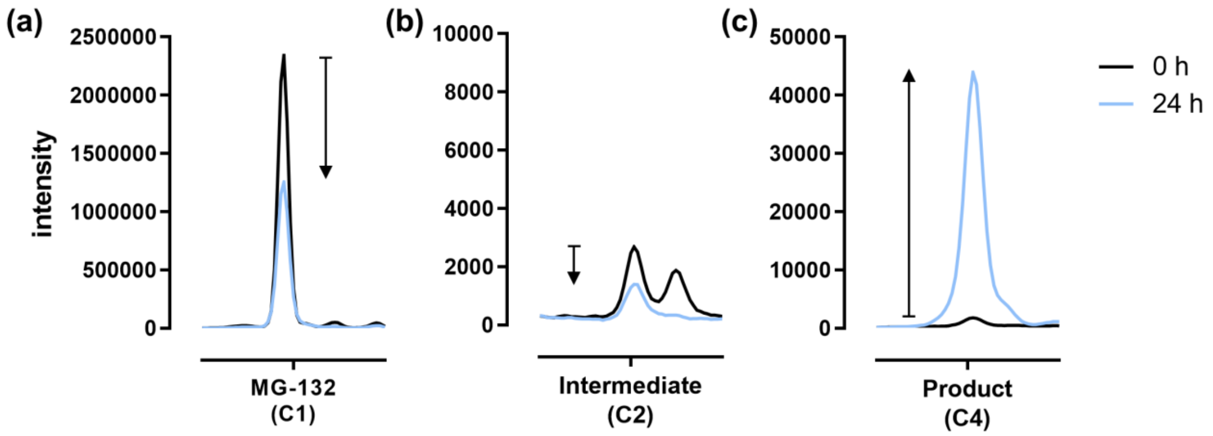

Supplementary Figure S5: Spectra of MG-132 (C1), intermediate (C2) and product (C4) of the reaction of MG-132 and L-cysteine in DMEM medium

MG-132 (100 µM) and L-Cys (L-cysteine; 200 µM) were incubated in DMEM medium for 24 h, and sampled at the indicated time points. (**a**) MG-132 (C1), (**b**) the intermediate (C2) and (**c**) the product (C4) were quantified via mass spectrometry (MS). The data provide background information to Fig 3.

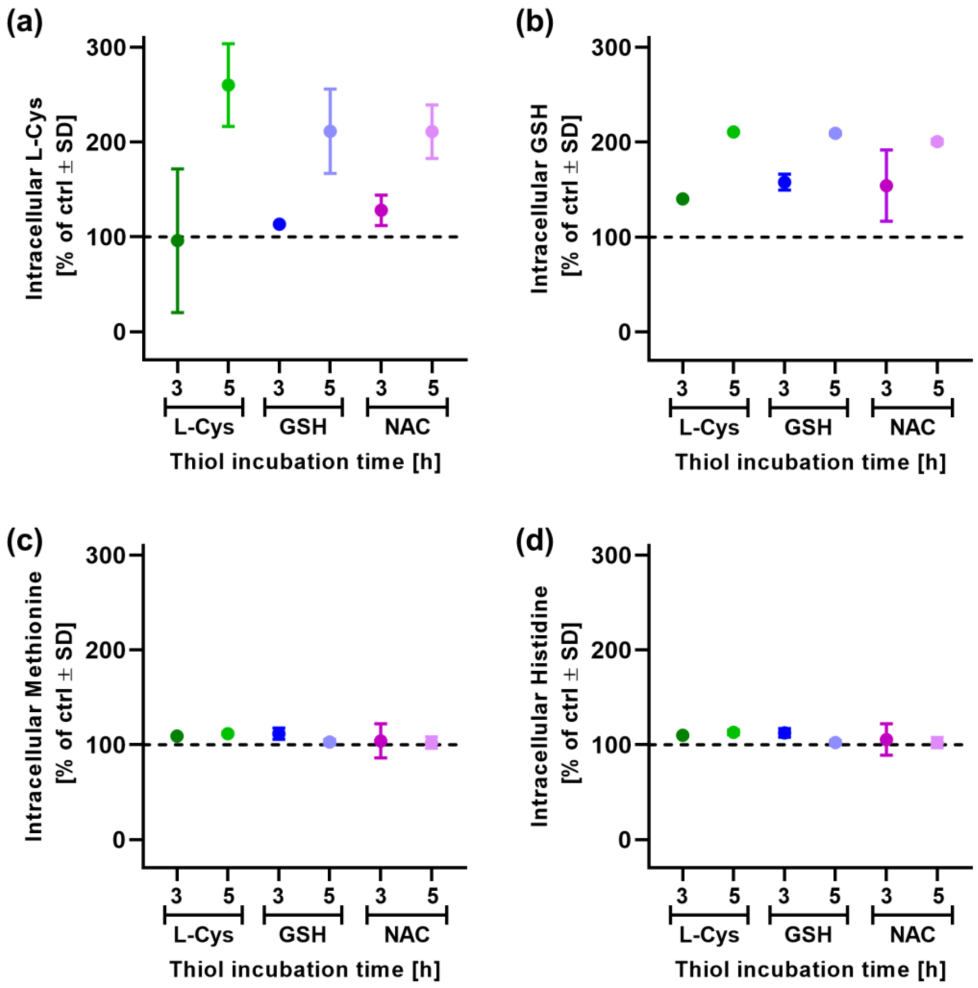

Supplementary Figure S6: Intracellular concentration of L-cysteine, glutathione, methionine and histidine after thiol incubation

Developing LUHMES neurons (d3) were incubated with 250 µM of the three thiols (L-cysteine: L-Cys; glutathione: GSH; N-acetyl-cysteine: NAC) for 3 h and 5 h. Then, cells were harvested to analyze the intracellular amino acid content. The intracellular amino acid concentrations were determined using the measured quantity in the cell fraction and the knowledge on the cellular volume (volume of 100’000 LUHMES cells: V = 150 ± 18 nl). All data is normalized to the untreated control and displayed as average of two replicates ± SD. (**a**) Upon thiol treatment, the intracellular L-Cys concentration increases up to 260% of the control level (untreated cells contained 6 ± 1 µM; dotted line). (**b**) The intracellular GSH concentration increased to up to 210% of the control level (untreated cells contained 906 ± 21 µM; dotted line). Other intracellular amino acids did not change. Methionine ((**c**); untreated cells contained

194 ± 7 µM; dotted line) and histidine ((**d**); untreated cells contained 200 ± 4 µM; dotted line) are shown as examples.

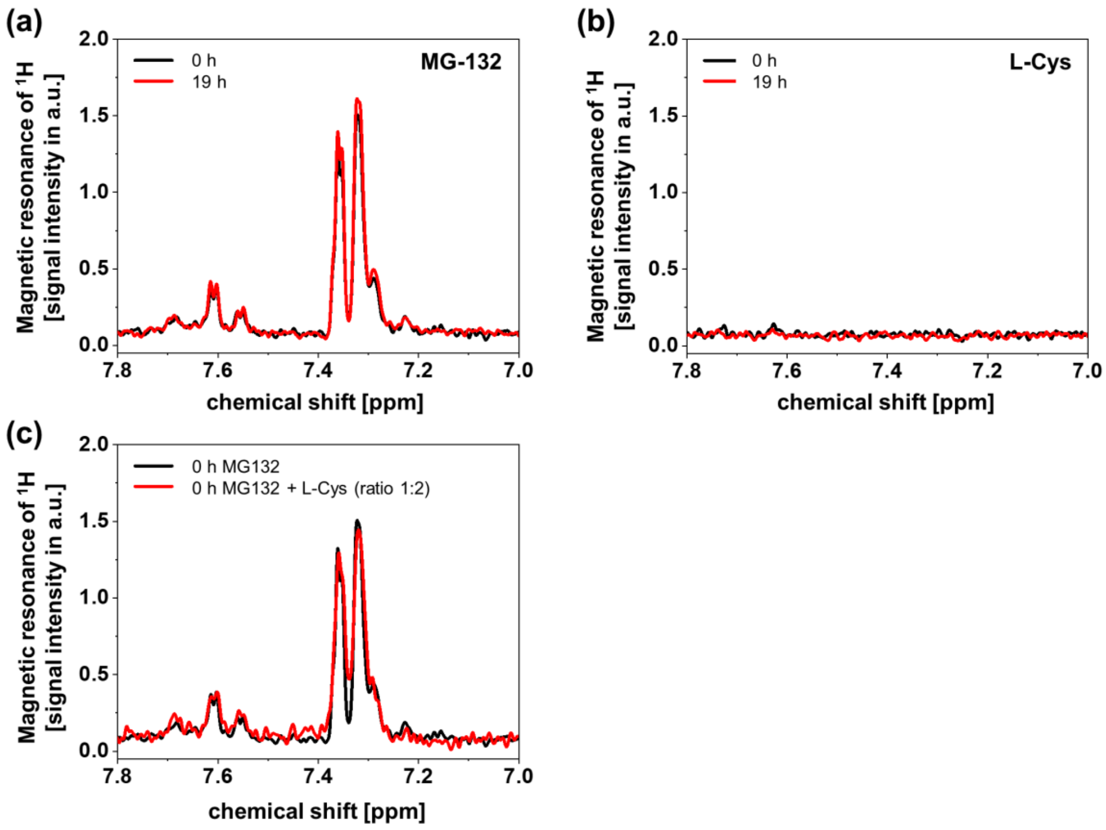

Supplementary Figure S7: ^{1}H-NMR-spectra of MG-132 and L-cysteine controls

This figure given background information on control conditions relevant to Fig 4. ^{1}H-NMR spectra were obtained at short intervals. (**a**) MG-132 (100 µM) was incubated for 19 h alone. No significant signal changes were observed in this time frame, indicating that MG-132 is stable under the experimental conditions. (**b**) L-Cys (L-cysteine; 200 µM) was incubated for 19 h alone. No signal was detected, indicating that L-Cys does not directly interfere with the quantification of the MG-132 reaction. (**c**) The spectrum of MG-132 alone is compared to the spectrum immediately after L-Cys addition. No significant signal change was observed. This confirms that L-Cys does not alter the MG-132 signal. Changes measured in Fig 4 are therefore to be considered to be due to the covalent reaction of MG-132 with L-Cys, not an artefact created by the presence of L-Cys.

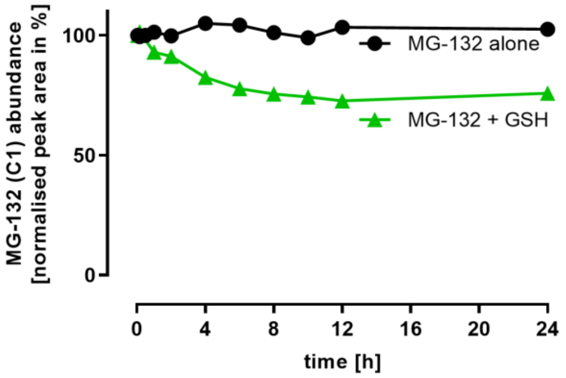

Supplementary Figure S8: Quantification of MG-132 (C1) in the presence or absence of GSH over the course of 24 h

MG-132 (100 µM) was incubated in DMEM medium for 24 h in the presence or absence of GSH (200 µM), and sampled at the indicated time points. MG-132 (C1) was quantified via mass spectrometry (MS).

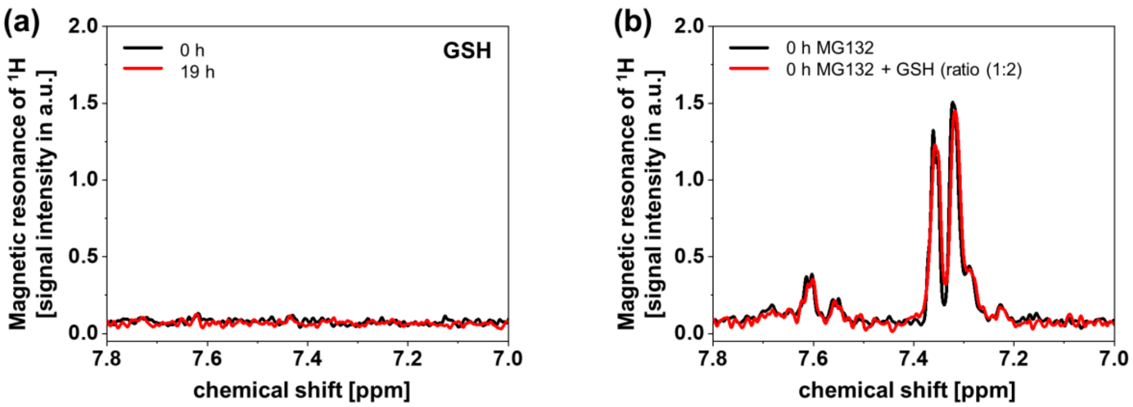

Supplementary Figure S9: ^{1}H-NMR-spectra of MG-132 and GSH

The figure displays the control data for the NMR data in Fig 5. (**a**) ^{1}H-NMR spectra were obtained for GSH (glutathione; 200 µM) alone. No signal was observed (at 0 or 19 h). Thus, GSH does not interfere with the quantification of the MG-132 reaction. (**b**) The spectrum of MG-132 alone is compared to the spectrum obtained immediately after GSH addition. No significant signal differences were observed. This indicates that GSH addition does not directly alter the MG-132 signal.

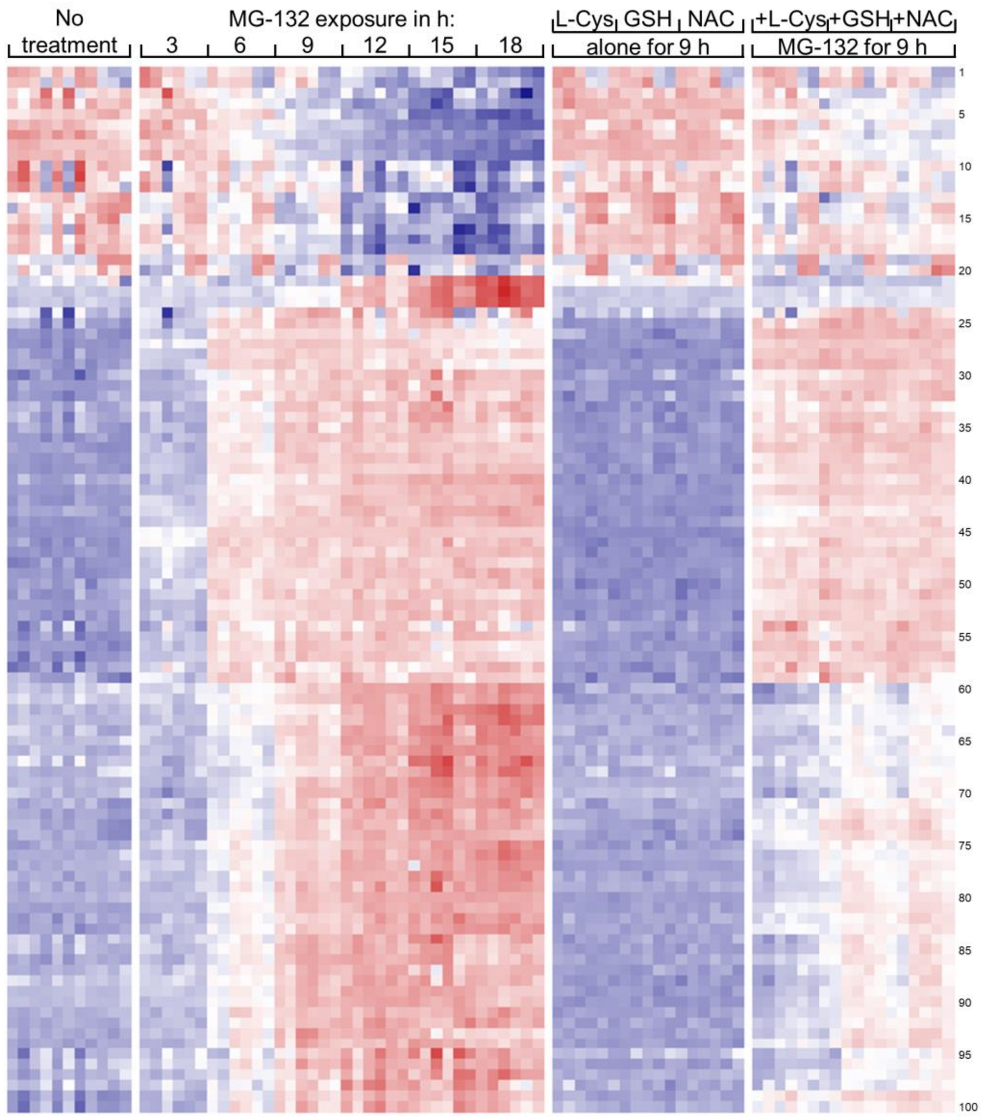

Supplementary Figure S10: Differential effects of thiols on gene expression changes by MG-132 (Top 100 DEGs)

A transcriptome analysis was performed on differentiated LUHMES neurons (d6), treated with MG-132 (100 nM) in the presence or absence of thiols (100 µM; L-Cys: L-cysteine; GSH: glutathione; NAC: N-acetyl-cysteine), as well as with the thiols alone. The color code indicates the row-wise z-score (full red: 1; white: 0; full blue: -2). The gene names (selected row numbers for orientation) are top to bottom: CLPTM1L (1), H2AC14, CTNNB1, TMSB10, PEG10 (5), PLCH2, RAB26, ST18, GRIA2, ZNF28 (10), ABRACL, TCF7L2, ALCAM, PRPH, PCP4 (15), GSTA4, SNCG, PPIAL4A, MYH3, NEFM (20), TNC, FLNC, TNFRSF10B, RAB39A, PSMA1 (25), ANP32E, PLAA, PSMD1, MAP1A, CYB5R1 (30), PALM3, ALDOA, NQO1,

BSCL2, SOD1 (35), UFD1, PSMB4, BAG2, GSR, UCHL1 (40), SCPEP1, EPDR1, XPOT, FAM219A, GCLM (45), BFR2, PSMC2, PSMD12, NPLOC4, SLC38A6 (50), SLMAP, ME1, USP14, PSMB2, PSMD14 (55), DAP3, KCTD20, VCP, NEFH, CBS (60), ALDH1L2, PPP1R15A, HMOX1, VEGFA, ATF5 (65), SLC7A3, FTH1, HSPB1, TRIB3, CBX4 (70), HSPD1, HSP90AA1, HSP90AB1, AHSA1, JMJD6 (75), DDIT3, SLC3A2, IARS1, SESN2, SLC7A5 (80), HSPH1, CHORDC1, ZFAND2A, ASNS, GPT2 (85), CTH, SLC7A1, YARS1, MTHFD2, GARS1 (90), CHAC1, SLC7A11, PSAT1, IFRD1, SARS1 (95), PHGDH, INHBE, NARS1, HSPA8, EIF5 (100). No obvious pattern of transcriptome changes was observed with the thiols alone.

Reference	PMID
How does conserved dopamine neurotrophic factor protect against and rescue neurodegeneration of PC12 cells?<br>Mei JM, Niu CS.	28852398
Inflammation kinase PKR phosphorylates α-synuclein and causes α-synuclein-dependent cell death.<br>Reimer L, Vesterager LB, Betzer C, Zheng J, Nielsen LD, Kofoed RH, Lassen LB, Bølcho U, Paludan SR, Fog K, Jensen PH.	29501855
Differential expression of PARK2 splice isoforms in an in vitro model of dopaminergic-like neurons exposed to toxic insults mimicking Parkinson's disease.<br>La Cognata V, Maugeri G, D'Amico AG, Saccone S, Federico C, Cavallaro S, D'Agata V.	28688199
Inhibition of store-operated calcium entry by sub-lethal levels of proteasome inhibition is associated with STIM1/STIM2 degradation.<br>Kuang XL, Liu Y, Chang Y, Zhou J, Zhang H, Li Y, Qu J, Wu S.	26960935
Synergistic stress exacerbation in hippocampal neurons: Evidence favoring the dual-hit hypothesis of neurodegeneration.<br>Heinemann SD, Posimo JM, Mason DM, Hutchison DF, Leak RK.	26934478
K(ATP) channel block prevents proteasome inhibitor-induced apoptosis in differentiated PC12 cells.<br>Nam YJ, Lee DH, Lee MS, Lee CS.	26142827
Benzodiazepinone derivatives protect against endoplasmic reticulum stress-mediated cell death in human neuronal cell lines.<br>Zou H, Limpert AS, Zou J, Dembo A, Lee PS, Grant D, Ardecky R, Pinkerton AB, Magnuson GK, Goldman ME, Rong J, Teriete P, Sheffler DJ, Reed JC, Cosford ND.	25544056
Proteasome activation is a mechanism for pyrazolone small molecules displaying therapeutic potential in amyotrophic lateral sclerosis.<br>Trippier PC, Zhao KT, Fox SG, Schiefer IT, Benmohamed R, Moran J, Kirsch DR, Morimoto RI, Silverman RB.	25001311
Nigrostriatal pathway degeneration in rats after intraperitoneal administration of proteasome inhibitor MG-132.<br>Wójcik S, Spodnik JH, Spodnik E, Dziewiątkowski J, Moryś J.	24729342
Preconditioning stimulus of proteasome inhibitor enhances aggresome formation and autophagy in differentiated SH-SY5Y cells.<br>Bang Y, Kang BY, Choi HJ.	24602982
N-Acetyl cysteine blunts proteotoxicity in a heat shock protein-dependent manner.<br>Jiang Y, Rumble JL, Gleixner AM, Unnithan AS, Pulugulla SH, Posimo JM, Choi HJ, Crum TS, Pant DB, Leak RK.	24096134
Astrocyte plasticity revealed by adaptations to severe proteotoxic stress.<br>Titler AM, Posimo JM, Leak RK.	23420451
Postnatal proteasome inhibition induces neurodegeneration and cognitive deficiencies in adult mice: a new model of neurodevelopment syndrome.<br>Romero-Granados R, Fontán-Lozano Á, Aguilar-Montilla FJ, Carrión ÁM.	22174927
Cytoplasmic accumulation and aggregation of TDP-43 upon proteasome inhibition in cultured neurons.<br>van Eersel J, Ke YD, Gladbach A, Bi M, Götz J, Kril JJ, Ittner LM.	21829535

Proteasome inhibition modeling nigral neuron degeneration in Parkinson's disease.<br>Xie W, Li X, Li C, Zhu W, Jankovic J, Le W.	20649845
Endogenous dopamine (DA) renders dopaminergic cells vulnerable to challenge of proteasome inhibitor MG132.<br>Zhou Z, Kerk S, Meng Lim T.	18484277
Protective effect against Parkinson's disease-related insults through the activation of XBP1.<br>Sado M, Yamasaki Y, Iwanaga T, Onaka Y, Ibuki T, Nishihara S, Mizuguchi H, Momota H, Kishibuchi R, Hashimoto T, Wada D, Kitagawa H, Watanabe TK.	19135031
A comparative study of proteasomal inhibition and apoptosis induced in N27 mesencephalic cells by dopamine and MG132.<br>Zafar KS, Inayat-Hussain SH, Ross D.	17504267
Proteasome inhibitor MG-132 induces dopaminergic degeneration in cell culture and animal models.<br>Sun F, Anantharam V, Zhang D, Latchoumycandane C, Kanthasamy A, Kanthasamy AG.	16870259
Proteasome mediates dopaminergic neuronal degeneration, and its inhibition causes alpha-synuclein inclusions.<br>Sawada H, Kohno R, Kihara T, Izumi Y, Sakka N, Ibi M, Nakanishi M, Nakamizo T, Yamakawa K, Shibasaki H, Yamamoto N, Akaike A, Inden M, Kitamura Y, Taniguchi T, Shimohama S.	14672949
Mitochondrial impairment triggers cytosolic oxidative stress and cell death following proteasome inhibition.<br>Maharjan S, Oku M, Tsuda M, Hoseki J, Sakai Y.	25077633
Ubiquitin-binding protein p62 expression is induced during apoptosis and proteasomal inhibition in neuronal cells.<br>Kuusisto E, Suuronen T, Salminen A.	11162503

Supplementary Figure S11: Studies utilizing MG-132 to model neurodegeneration

MG-132 is widely used to model neurodegeneration in cell culture and animal-based systems.

References are displayed with title, authors and PMID.

Supplementary References

1. Delp, J.; Gutbier, S.; Cerff, M.; Zasada, C.; Niedenführ, S.; Zhao, L.; Smirnova, L.; Hartung, T.; Borlinghaus, H.; Schreiber, F.; et al. Stage-specific metabolic features of differentiating neurons: Implications for toxicant sensitivity. *Toxicol Appl Pharmacol* **2018**, *354*, 64-80, doi:10.1016/j.taap.2017.12.013.
2. Suciu, I.; Delp, J.; Gutbier, S.; Ückert, A.K.; Spreng, A.S.; Eberhard, P.; Karreman, C.; Schreiber, F.; Madjar, K.; Rahnenführer, J.; et al. Dynamic Metabolic and Transcriptional Responses of Proteasome-Inhibited Neurons. *Antioxidants (Basel)* **2023**, *12*, doi:10.3390/antiox12010164.
3. Stiegler, N.V.; Krug, A.K.; Matt, F.; Leist, M. Assessment of chemical-induced impairment of human neurite outgrowth by multiparametric live cell imaging in high-density cultures. *Toxicol Sci* **2011**, *121*, 73-87, doi:10.1093/toxsci/kfr034.
